# *mzGroupAnalyzer*-Predicting Pathways and Novel Chemical Structures from Untargeted High-Throughput Metabolomics Data

**DOI:** 10.1371/journal.pone.0096188

**Published:** 2014-05-20

**Authors:** Hannes Doerfler, Xiaoliang Sun, Lei Wang, Doris Engelmeier, David Lyon, Wolfram Weckwerth

**Affiliations:** Department of Ecogenomics and Systems Biology, University of Vienna, Vienna, Austria; The George Washington University, United States of America

## Abstract

The metabolome is a highly dynamic entity and the final readout of the genotype x environment x phenotype (GxExP) relationship of an organism. Monitoring metabolite dynamics over time thus theoretically encrypts the whole range of possible chemical and biochemical transformations of small molecules involved in metabolism. The bottleneck is, however, the sheer number of unidentified structures in these samples. This represents the next challenge for metabolomics technology and is comparable with genome sequencing 30 years ago. At the same time it is impossible to handle the amount of data involved in a metabolomics analysis manually. Algorithms are therefore imperative to allow for automated *m/z* feature extraction and subsequent structure or pathway assignment. Here we provide an automated pathway inference strategy comprising measurements of metabolome time series using LC- MS with high resolution and high mass accuracy. An algorithm was developed, called *mzGroupAnalyzer*, to automatically explore the metabolome for the detection of metabolite transformations caused by biochemical or chemical modifications. Pathways are extracted directly from the data and putative novel structures can be identified. The detected *m/z* features can be mapped on a van Krevelen diagram according to their H/C and O/C ratios for pattern recognition and to visualize oxidative processes and biochemical transformations. This method was applied to *Arabidopsis thaliana* treated simultaneously with cold and high light. Due to a protective antioxidant response the plants turn from green to purple color via the accumulation of flavonoid structures. The detection of potential biochemical pathways resulted in 15 putatively new compounds involved in the flavonoid-pathway. These compounds were further validated by product ion spectra from the same data. The *mzGroupAnalyzer* is implemented in the graphical user interface (GUI) of the metabolomics toolbox COVAIN (Sun & Weckwerth, 2012, Metabolomics 8: 81–93). The strategy can be extended to any biological system.

## Introduction

Metabolomic techniques have been recently established and refined to characterize the widely heterogeneous small molecules present at a specific time in a living tissue. Several analytical approaches exist for the application of metabolomics to various biological questions, for instance gas chromatography coupled to mass spectrometry (GC-MS) for the detection of small and volatile compounds [1] or capillary electrophoresis coupled to mass spectrometry (CE-MS) for charged compounds [2]. The method with the highest preference for larger and more hydrophobic metabolites and complementary to GC-MS is liquid chromatography coupled to mass spectrometry (LC-MS) [3], [4]. Recently, we showed that the GC-MS and LC-MS techniques can be integrated into a combined platform to increase the total coverage of the metabolome, as well as to provide insights into the mutual regulation of both primary and secondary metabolism by analysis of the same sample and subsequent data merging and processing [4]–[6]. Metabolomics-via-LC-MS approaches have yet to hit the ranks of other analytical techniques with respect to their robustness and database availability, but no other method has the potential to achieve a better coverage of the metabolome whilst maintaining high resolution and inferring additional structural information in the process. In contrast to metabolite profiling on the GC-MS platform, where standard operating procedures and large databases are present and being improved continuously, there exists little standardization on how to approach the analysis of larger metabolites using LC-MS [7]. A significant step forward in the field of untargeted metabolomics is the advent of instruments capable of sub-ppm mass accuracy measurements as well as precursor fragmentation, enabling the acquisition of the exact mass as well as obtaining molecule fragment information in order to construct a meaningful sum formula [8], [9]. Owing to these technological advances, metabolomics has already proven to be a valuable tool in fields like biomarker discovery and functional genomics [10]–[12]. In this study, we introduce an algorithm called *mzGroupAnalyzer* to provide characterization and identification of data signals acquired by an LC-Orbitrap-FT-MS system utilizing sub-ppm mass measurements and intelligent sum formula query. We applied this strategy to plant secondary metabolism. Plants are most important resources for natural products, providing the highest diversity of chemical structures in the range of 200.000–400.000 different compounds and a high *in vivo* plasticity in response to environmental conditions. Due to this diversity of chemical structures ranging from simple hydrocarbons to complex heteroatomic molecules, the elucidation of the metabolome of higher plants and in general of natural products of any origin poses a challenge for metabolomic techniques. Traditionally, the metabolism of higher plants is subclassified into the primary metabolism, which comprises molecules with a low molecular weight that are involved in the central energy conversion cycles of the organism, and the secondary metabolism, which is not per se involved in energy homeostasis but rather involved in the chemical communication with the environment. Only recently, the field of plant secondary metabolites has begun moving more and more into the focus of new bio-analytical techniques and their stereotypic role as simple chemical weapons is being revised as numerous findings indicate their ability to stimulate vital processes in the cell by regulating the concentration of reactive oxygen species *in vivo* [13], [14]. Also, secondary metabolites are of vast interest to the area of medicine and nutrition, as these phytochemicals often possess physiological activity in the human body, for example antioxidant activity or cancer chemoprevention [15].

To provide a suitable experimental system, we stressed *Arabidopsis thaliana* plants by parallel cold and light treatment introducing high oxidative stress over a 3-week interval. This led to a comprehensive switch of the vegetative growth metabolism to protective accumulation of secondary metabolites. We show a way of embedding the multitude of signals into a biochemical context through automated detection of metabolic steps between the acquired *m/z* features. Putative structures are inferred by analysis of the product ions from the same data. By applying *mzGroupAnalyzer* to LC-MS raw data, we are able to prove the existence and relation of known molecules as well as propose novel compounds and pathways, in the present case molecules arising during oxidative stress within the secondary metabolism of *Arabidopsis thaliana*. This strategy can be applied systematically and conveniently to any kind of LC-MS data set and is expected to improve identification and structural elucidation in complex metabolomics data, which is currently the limiting step in large-scale metabolomics studies.

## Materials and Methods

### Plant material and harvest

*Arabidopsis thaliana* Col-0 was cultivated in a growth chamber under controlled conditions: light intensity was 280 µmol m^−2^ s^−1^ in an 8-hour light/16-hour dark day cycle; relative humidity was 60% with an average temperature of 22°C. Time point “zero” of cold stress consisted of replicates of non-stressed plants, while every 2 days another sample batch of cold-acclimated plants in a 4°C cold chamber was taken. Rosette leaves were harvested approximately 2 hours after the beginning of the light period. Metabolic activity in the leaves was quenched by immediately putting the plant material into liquid nitrogen after harvesting. Deep-frozen leaf material was ground to a fine powder with a pestle and mortar under steady addition of liquid nitrogen and subsequently stored at −80°C before measurement.

### Chemicals

Methanol (HPLC-grade), chloroform (anhydrous, >99%, p.a.) and acetonitrile (UHPLC-grade) were purchased from Sigma-Aldrich (Vienna, Austria). Formic acid (98–100%) was purchased from Merck (Vienna, Austria). Chloramphenicol (>98%) and Ampicillin trihydrate (analytical standard) were purchased from Fluka (Vienna, Austria).

### Extraction procedure and sample preparation for secondary metabolite analysis

For LC-MS analysis, about 50 mg of frozen plant-leaf material was extracted by 1 ml pre-chilled 80/20 v:v MeOH/H_2_O solution containing 1 µg of the internal standards Ampicillin and Chloramphenicol. Samples were centrifuged at 15.000 g for 15 minutes. The supernatant was dried out overnight in a new tube and re-dissolved in 100 µl of 50/50 v:v MeOH/H_2_O solution and centrifuged again for 15 minutes at 20.000 g. The supernatant was then filtered through a STAGE tip (Empore/Disk C18, diameter 47 mm) before it was conveyed into a GC vial with a micro insert tip. Plant extracts were further extracted with 500 µl of chloroform to remove the highly abundant lipid components. The LC-MS method for secondary metabolite analysis has been described before [6].

### Data processing, *mzGroupAnalyzer* and pathway viewer

Both *mzGroupAnalyzer* and *Pathway Viewer* have been integrated into the GUI of the COVAIN toolbox [16]. The standalone version of COVAIN can be downloaded at http://www.univie.ac.at/mosys/software.html. The data processing strategy and subsequent analysis of the data using *mzGroupAnalyzer* and *Pathway viewer* are explained in the *mzGroupAnalyzer*-Tutorial (Figure S1). m/z-values acquired by LC-MS were exported to Excel data sheets using the Xcalibur software. Elemental composition determination was enabled, with a maximum of 10 possible sum formulas for each compound and a ppm deviation of 1.

The single excel-sheets can be uploaded to *mzGroupAnalyzer* via the GUI of COVAIN. Furthermore, a user-defined rules-file is uploaded and the folder for storage of the result-files is provided. By starting the *mzGroupAnalyzer*, the lists of *m/z* values with associated chemical compositions from Xcalibur output are read and the atomic differences between m/z-pairs are calculated and compared with the putive chemical modifications provided by the rules-file.

Based on all chemical modifications provided by the rules-file, *mzGroupAnalyzer* searches pathways between pairs of *m/z* features. Regarding each *m/z* feature as a node in a metabolic network, an edge connects two nodes if a chemical modification exists. These edges and nodes generate large networks. A pathway can therefore be constructed by searching the shortest path between two nodes. Redundant paths that are included in other longer paths are removed. The pathway searching algorithm can deal with time series data by filtering out pathways that do not reflect the correct chronological order of the given measurements. For example, there is no path from *m/z* feature *A* occurring on Day 2 to *m/z* feature *B* occurring on Day 1, although theoretically a chemical modification from *A* to *B* is possible. Finally, to better visualize the pathways, we further developed *Pathway Viewer*, which is integrated in *mzGroupAnalyzer*, and is able to plot the pathways after a series of user-defined filtering options like *m/z* range or time points. The following result files will be created, exported and saved:

1. Transformations corresponding to the rules-file.
2. A ranking of frequently found chemical modifications.
3. Putative transformations that were not listed in the rules-file.
4. A mzStructure file for *Pathway viewer*.
5. A Pajek-file (http://vlado.fmf.uni-lj.si/pub/networks/pajek/) for network visualization.

**Figure 1:**
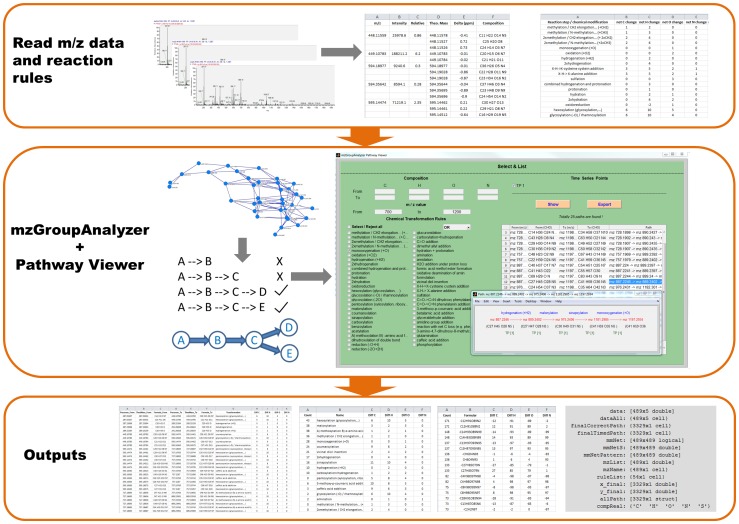
Scheme of the *mzGroupAnalyzer* and *Pathway Viewer* algorithm and GUI implementation. The program reads the *m/z* features which are extracted from Xcalibur, as well as the user predefined reaction rules. Then it finds transformations between all pairs of *m/z* features, and reports the frequency of transformations for the listed and not listed but potentially existing rules. Next, the program starts searching pathways inside the *m/z* features' network. A shorter path existing in other longer paths is removed, thereby non-redundant pathways are obtained. Then, *mzGroupAnalyzer* opens the Pathway Viewer, in which pathways satisfying user-defined filtering options will be displayed on the panel. The pathway diagram, which consists of reaction rules, *m/z* feature names, compositions and time points, can be plotted by clicking the table. Finally, all the results, including the frequency table of transformations, the interconnected network visualization file (in Pajek's format), the inferred pathways and a Matlab workspace (suffixed with mzStruct.mat) containing all results, will be exported to the user-specified folder.

Next the *Pathway viewer* is started and a table of transformations and the possibility for visualization of pathways is provided (see Figure S1 – *mzGroupAnalyzer*-Tutorial). The above process is summarized in Figure 1. The list of chemical and biochemical reactions searched by *mzGroupAnalyzer* (currently comprising 56 metabolic reaction steps in the provided rules-file) is shown in Table S1. This list can be easily extended to novel transformations.

For the construction of the van Krevelen plots, sum formulas with *mzGroupAnalyzer*-predicted metabolic transformations were assorted in an Excel sheet and their O/C and H/C ratios were calculated. These values were exported to SigmaPlot 12.3 and mapped to a multiple scatter plot within the boundaries 0 to 1 for the O/C ratio and 0 to 2 for H/C ratio.

## Results and Discussion

### Development of the *mzGroupAnalyzer* algorithm to identify biochemical and chemical transformations in non-targeted metabolomics data

**Figure 2:**
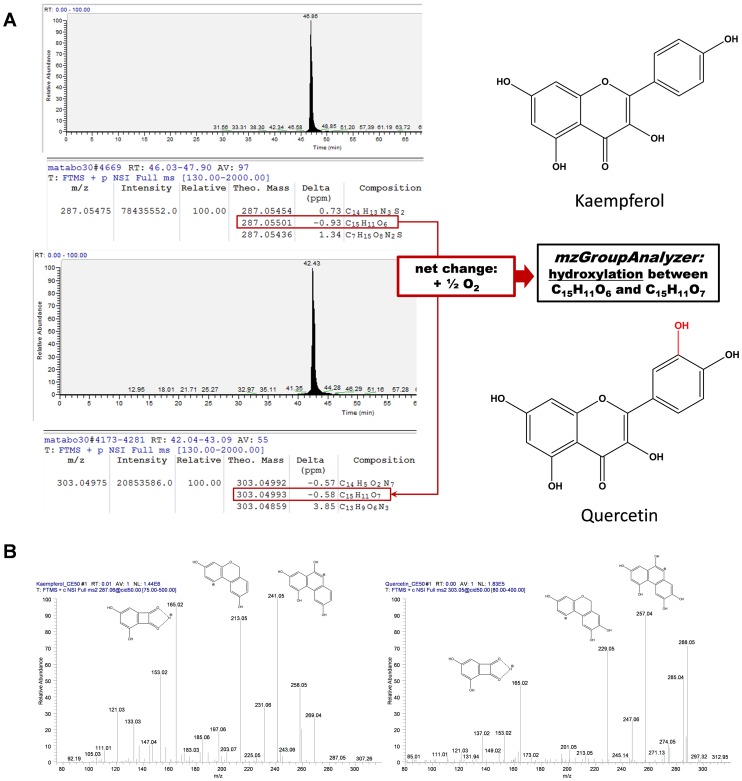
*mzGroupAnalyzer* is able to detect possible metabolic steps out of various proposed sum formulas for a measured *m/z* feature. **A** Kaempferol and Quercetin standards measured by LC-MS result into several sum formula suggestions for the measured mass-to-charge-ratio (*m/z*). Because a low ppm deviation of assigned elemental composition to the mass is not the decisive factor, the correct sum formula might not be the first one proposed and thus several must be looked at, which is handled by the program automatically. If *mzGroupAnalyzer* finds a possible reaction step (out of a list of reactions which can be altered manually), it is reported to the user. **B** MS^2^ spectra of Kaempferol (left) and Quercetin (right). The fragmentation schemes are in accordance with published literature [42], [43]. The difference of one oxygen atom (nominal mass 16) is visible in the fragments *m/z* 213→229 and 241→257, while *m/z* 165 occurs in both product scans.

The development and application of algorithms is essential to systematically search for biochemical and chemical transformations of compounds and to find putative pathways in highly complex LC-MS based metabolomics data. We have established an algorithm which is able to extract putative chemical transformations from high mass accuracy metabolome data. The algorithm generates multiple potential pathways directly from raw-LC-MS data and visualizes these as networks. The entire approach is implemented as a graphical user interface (GUI) in COVAIN (Figure 1). COVAIN is a toolbox for statistical data mining in metabolomics and other OMICS approaches [16] in the Matlab environment. In order to evaluate the algorithm, we measured various reference compounds of typical plant secondary metabolites with nano-UPLC-Orbitrap-MS. Subsequently, sum formulas were generated in Xcalibur 2.0 using the integrated sum formula calculator. Within a 1 ppm mass accuracy window and using the monoisotopic masses of several common elements, possible sum formulas were obtained (Figure 2 and Figure S1 – *mzGroupAnalyzer*-Tutorial). Many of the sum formulas did not result in feasible chemical structures but had a very high mass accuracy according to the measured compound, thus leading to false positives. As a consequence the correct sum formula prediction was not among the top first hits. The fact that high mass accuracy alone is not sufficient for correct sum formula prediction has been shown before [17], [18]. This is especially true for metabolites which often contain elements like S and P, or even halogens. The utilization of electrospray ionization tends to produce Na^+^, K^+^ and other adducts in positive ionization mode. Because *mzGroupAnalyzer* looks for putative chemical transformations of compounds the considered number of sum formulas can be reduced. The application of *mzGroupAnalyzer* revealed metabolic conversions of the protonated reference compound such as the addition of a hydroxyl group to Kaempferol leading to Quercetin (Figure 2). Due to this chemical transformation the sum formula pair of Kaempferol and Quercetin is automatically detected. Indeed, this reaction occurs in the flavonol biosynthetic pathway catalyzed by a flavonoid 3′-monooxygenase. The constraints for detectable metabolic reactions (e.g.+1C+2H denotes a net methylation) are uploaded to the program before performing the analysis and can be customized by the user (Figure S1 – *mzGroupAnalyzer*-Tutorial). *mzGroupAnalyzer* is also able to recognize the frequency of equidistant steps between *m/z* values and exports these data as suggestions for novel modifications (see Methods section). Furthermore, whole series of reaction steps in the data can be detected and analyzed in the context of metabolic pathways. This will be discussed in the next sections.

### Cold- and light-induced stress has a dramatic effect on the *Arabidopsis thaliana* metabolome

**Figure 3:**
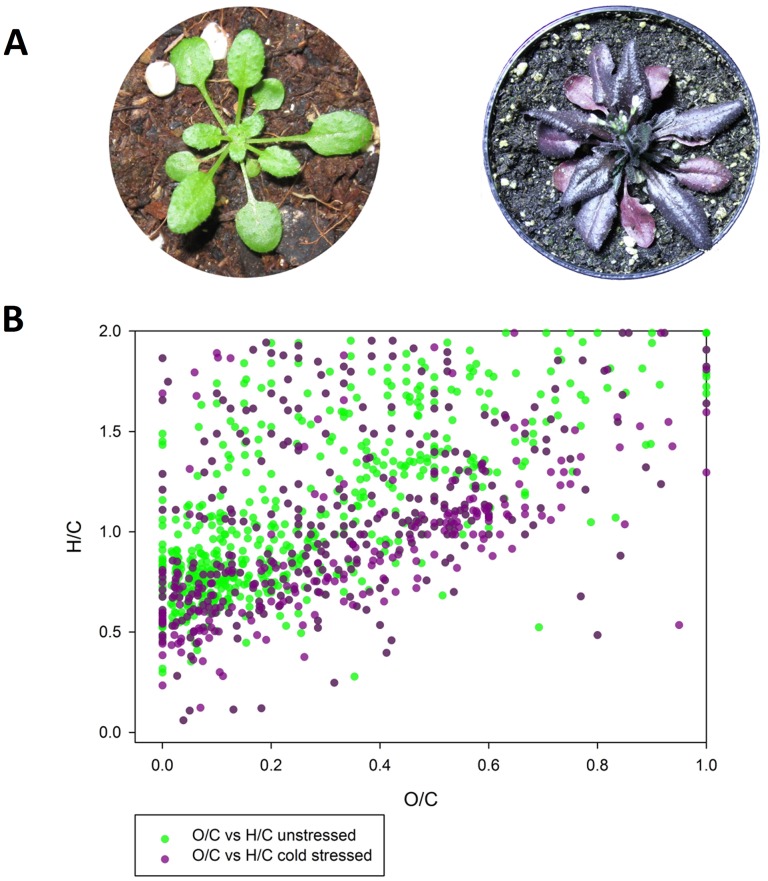
After oxidative stress the *Arabidopsis thaliana* plants turn from green into purple indicating a dramatic shift in metabolism, specifically elevated flavonoid biosynthesis involved in oxidative stress protection [6]. **A** Plants turns from green to purple under high light and cold temperature treatment. **B** Van Krevelen diagram of the most abundant *m/z* values of unstressed (green dots) and 20-day cold stressed (purple dots) Arabidopsis plants. A clear shift of metabolism in the stressed plants is visible.

To test the performance of the *mzGroupAnalyzer* algorithm we designed the following experiment. *Arabidopsis thaliana* plants were exposed to excess irradiation in a 4°C environment. This treatment is described in the literature to produce high levels of reactive oxygen species (ROS) [19]–[21]. ROS, such as hydrogen peroxide (H_2_O_2_), hydroxyl radicals (OH), superoxide anion (O_2_^−^) and singlet oxygen (^1^O_2_), are unavoidable by-products of photosynthetic organisms occurring in organelles with a high oxidative turnover rate during normal metabolic activity and can be highly damaging for cells and tissues under stress [13], [22], [23]. These stress conditions require an effective scavenging system in order to prevent the organism from being damaged by free radicals [24]. Especially biomolecules from the phenylpropanoid family, such as flavonoids and anthocyanins, have been recognized as effective radical-scavenging compounds [25], [26]. After several days of stress in our experimental systems, the plants began to produce violet pigments in the rosette leaves (Figure 3A). The purple color is the result of the accumulation of anthocyanidins as a response to oxidative stress [25]–[28]. The enhanced production of anthocyanidins under these stress conditions requires a large-scale metabolic reprogramming as recently described by us [6]. Leaf extracts of the cold/light-treated *Arabidopsis thaliana* plants were analyzed with LC/MS. From these analyses sum formulas of putative metabolites were generated based on the acquired *m/z* values focusing only on C, H and O elements (see also Figure S1 – *mzGroupAnalyzer*-Tutorial). The generated sum formulas were loaded into a van Krevelen plot to visualize the chemical and biochemical transformations of metabolites during cold and light stress (Figure 3B). Oxidative shifts in the plot induced by cold-related stress might be explained by the incorporation of oxygen, as well as radical scavenging by redox-active compounds, such as aromatic hydroxyl groups which can stabilize radicals after deprotonation. Van Krevelen plots were originally introduced to characterize carbon-based resources like mineral oils and coal according to their possible chemical composition acquired by high-resolution mass spectrometry [29], [30]; only recently, van Krevelen plots have been applied as useful tools for visualization of metabolic processes and pathways [31] as well as for sum formula annotation of natural organic matter (NOM) [32].

**Figure 4:**
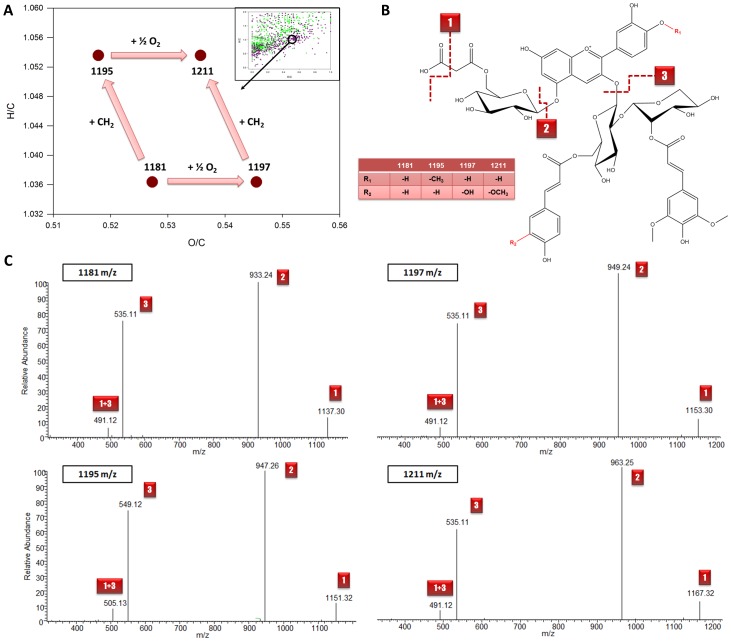
Exploration of the van Krevelen diagram created by sum formulas with chemical transformations detected by *mzGroupAnalyzer*. **A** *m/z* 1181, 1195, 1197 and 1211 are interconnected with net shifts of ½ O_2_ and a CH_2_ group and form a rhombic pattern. **B** Proposed fragmentation scheme of these compounds under the chosen conditions. **C** Product ion scans show similar fragmentation behavior of the polysubstituted anthocyanins. The spectrum of *m/z* 1195 shows a peak at *m/z* 549, pointing to a methyl group at the cyanidin core. A putative methylation site is shown.

The investigation of van Krevelen plots composed of untargeted metabolomics data can validate structural familiarity between compounds through a metabolic pattern as depicted in Figure 4. In the O/C ratio range from 0.51 to 0.55 and H/C range of 1.036 to 1.056, the chemical relation of compounds *m/z* 1181, 1195, 1197 and 1211 is visible (Figure 4A), while their structures are validated by MS^2^ product ion scans (Figure 4B and 4C). These compounds were also automatically detected by the *mzGroupAnalyzer* approach when investigating the putative chemical and biochemical transformations from the generated sum formula hits (see also Figure S1 – *mzGroupAnalyzer*-Tutorial). In the following section we explored the potential of *mzGroupAnalyzer* to reveal full pathways leading to novel structures.

### Broad-scale analysis of metabolic conversions and novel structure prediction by *mzGroupAnalyzer* in the cold/light stress metabolome of *Arabidopsis thaliana*

Time-dependent sampling of *Arabidopsis thaliana* leaf samples in cold stress yielded strong alterations in the metabolic profiles (see above). All precursor ions from the LC/MS analysis obtained from one sampling time point were assigned to ten possible sum formulas with the following parameters: 100 max C, 200 max H, 50 max O, 10 max N, 1 ppm maximum deviation from suggested sum formula. Data from all time points were exported from Xcalibur 2.0 software as single Excel sheets and uploaded into *mzGroupAnalyzer* using the graphical user interface (see above and methods; see Figure S1 – *mzGroupAnalyzer*-Tutorial). Further, a user-defined rules-file of the molecular shifts of chemical and biochemical transformations needs to be uploaded via the GUI. Here, we provide a rules-file with 56 reactions. By starting the *mzGroupAnalyzer* via the GUI, the algorithm detects metabolic transformations between pairs of *m/z* values in the uploaded list of suggested sum formulas. A table of detected pathways is generated using the GUI option “Pathway Viewer”. Individual pathways can be visualized by clicking on the corresponding cell. Furthermore, the whole pathway network can be exported as a Pajek-file for visualization (see Figure S1 – *mzGroupAnalyzer*-Tutorial and Figure 1). Over the course of the cold and light stress, several highly frequent reactions were identified. Overall, the 10 predominant reactions were mono-oxygenations, followed by hydrogenations, hydrations, methoxylations, methylations, oxidoreductions, oxidations, malonylations, hexosylations, and dihydroxylations of double bonds. Due to possible false positives within the detected metabolic steps and reactions, these reaction lists have to be carefully validated manually and present preliminary results. More evaluation of the *mzGroupAnalyzer* and further proof-of-concept studies are needed in the future.

**Figure 5:**
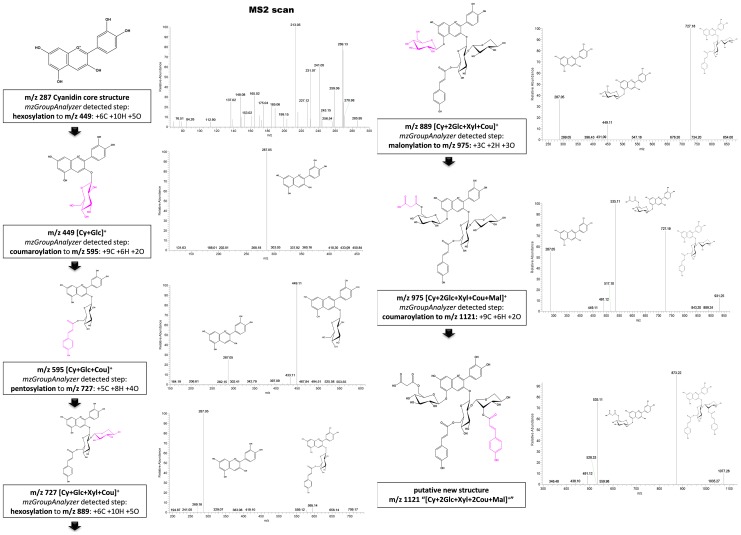
Identification of biochemical transformations of *in vivo* data using *mzGroupAnalyzer*. A metabolic pathway leading to a putative new compound *m/z* 1121 is revealed. Amongst several hundreds of other interlinked *m/z* values in the data, the figure shows metabolic transitions derived from sub-ppm accuracy measurements on the left side and their corresponding MS^2^ product ion scans on the right. Comparison of the spectral information from step to step reveals the possible location of metabolic structural changes. Stereochemistry is assumed due to literature findings [33].

Nevertheless, *mzGroupAnalyzer* reported many reactions, which were validated to be metabolic pathways: By investigating the information from the MS^2^ spectra as well as comparing them with published literature (e.g. [33], see Table 1), the presence of several compounds of the anthocyanin family in the *Arabidopsis* plants was revealed. Additionally, the program reported metabolic steps from these compounds to previously undescribed *m/z* features, which are suspected to be new anthocyanins. In Figure 5, it is shown how a metabolic pathway is extracted by *mzGroupAnalyzer*. The prediction of the pathway is validated by corresponding MS^2^ product ion spectra from the same data set. This pathway leads to a putatively novel compound *m/z* 1121 which was detected automatically by *mzGroupAnalyzer*:

**Table 1:** Putative compounds including their *mzGroupAnalyzer*- predicted sum formulas, the corresponding exact mass as well as dominant MS^2^ product ion fragments.

name or *m/z* value	sum formula detected by *mzGroupAnalyzer*	exact mass	main fragments	mass accuracy (ppm)	reference
A1	C_32_H_39_O_20_	743.20292	287, 581	0.35	Tohge et al. [33]
A2	C_35_H_41_O_23_	829.20331	287, 535, 581, 785	0.29	Tohge et al.
A3	C_41_H_45_O_22_	889.23970	287, 449, 727	−0.11	Tohge et al.
A4	C_43_H_49_O_24_	949.26083	287, 449, 787	0.48	Tohge et al.
A5	C_44_H_47_O_25_	975.24009	491, 535, 727, 931	−0.23	Tohge et al.
A6	C_47_H_55_O_27_	1051.29252	449, 889	−0.27	Tohge et al.
A7	C_52_H_55_O_26_	1095.29761	449, 933	0.41	Tohge et al.
A8	C_50_H_57_O_30_	1137.29292	491, 535, 889	−0.13	Bloor & Abrahams [40]
A9	C_55_H_57_O_29_	1181.29800	491, 535, 933, 1137	−0.28	Bloor & Abrahams
A10	C_58_H_65_O_31_	1257.35043	449, 1095	−0.07	Tohge et al.
A11	C_61_H_67_O_34_	1343.35083	491, 535, 1095, 1299	0.51	Bloor & Abrahams
A12	C_26_H_29_O_15_	581.15010	287, 449	0.43	Tohge et al.
A13	C_35_H_35_O_17_	727.18688	287, 581	0.23	Tohge et al.
A14	C_37_H_39_O_19_	787.20801	287	0.21	Tohge et al.
A15	C_41_H_45_O_22_	889.23970	287	−0.14	Tohge et al.
A16	C_46_H_45_O_21_	933.24478	287, 727	0.28	Tohge et al.
A17	C_52_H_55_O_26_	1095.29761	449, 933	0.56	Tohge et al.
919	C_42_H_47_O_23_	919.25026	287, 757	0.16	-
991	C_44_H_47_O_26_	991.23501	491, 535, 743, 947	0.17	-
1005	C_45_H_49_O_26_	1005.25066	491, 535, 757, 961	0.28	-
1035	C_46_H_51_O_27_	1035.26122	491, 535, 787, 991	0.31	-
1065	C_51_H_53_O_25_	1065.28704	449, 903	0.29	-
1081	C_51_H_53_O_26_	1081.28196	449, 919	0.68	-
1111	C_52_H_55_O_27_	1111.29252	449, 949	0.36	-
1121	C_53_H_53_O_27_	1121.27687	491, 535, 873, 1077	0.13	-
1125	C_53_H_57_O_27_	1125.30817	449, 963	0.51	Saito et al. [39]
1151	C_54_H_55_O_28_	1151.28744	491, 535, 903, 1107	0.48	-
1167	C_54_H_55_O_29_	1167.28235	491, 535, 919, 1123	0.54	Kasai et al. [41]
1195	C_56_H_59_O_29_	1195.31365	505, 549, 947, 1151	0.49	-
1197	C_55_H_57_O_30_	1197.29292	491, 535, 949, 1153	0.28	Saito et al.
1211	C_56_H_59_O_30_	1211.30857	491, 535, 963, 1167	0.05	Saito et al.
1313	C_60_H_65_O_33_	1313.34026	491, 535, 1065, 1269	0.13	-
1329	C_60_H_65_O_34_	1329.33518	491, 535, 1081, 1285	0.43	-
1359	C_61_H_67_O_35_	1359.34574	491, 535, 1111, 1315	0.58	-
1373	C_62_H_69_O_35_	1373.37156	491, 535, 1125, 1329	0.67	-
1549	C_72_H_77_O_38_	1549.40873	535, 1301, 1505	0.57	-

The nomenclature is according to [33]. Compounds *m/z* 1125, 1197 and 1211 were found in *Matthiola incana* by [39].

**Figure 6:**
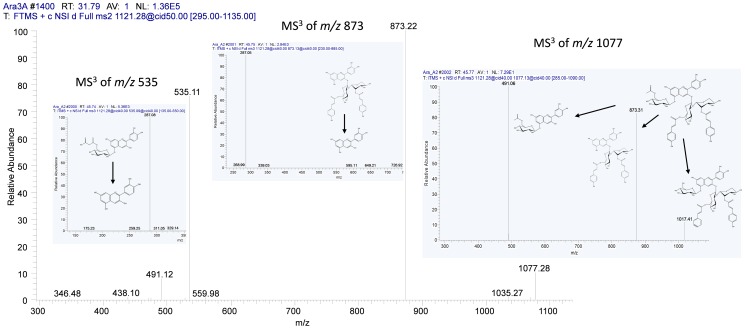
Structure validation of *m/z* 1121 by MS^3^ product ion scans. Both MS^2^ fragments *m/z* 535 and 873 result in the core cyanidin structure by undergoing MS^3^ fragmentation. *m/z* 1077, the putatively decarboxylized form of 1121, yields *m/z* 491, as observed in the MS^2^ spectrum already, by scission of the 3-O-glycosidic bond. Fragment *m/z* 873 arises again from the breaking of the glycosidic bond at 5-O. *m/z* 1017 would comply with the complete removal of the rest of the former malonyl group together with a water loss (−60 u). A putative structure is given.

*mzGroupAnalyzer* detected reactions between the *m/z* signals 287, 449, 595, 727, 889, 975 and 1121. *m/z* 287, the core cyanidin structure, is hexosylated to structure *m/z* 449, which itself shows the intact cyanidin structure in its MS^2^ product ion scan and which has been reported as cyanidin 5-O-glycoside [33]. A coumaroyl group is then added to the sugar group in *m/z* 449, resulting in compound *m/z* 595, while showing the previous two compounds in the MS^2^ spectrum. To *m/z* 595, a 5-C sugar is then added to the already existing hexose group – judged from the MS^2^ spectrum – forming compound *m/z* 727. In the MS^2^ spectrum of *m/z* 727 (“A13”) only *m/z* 595 is visible, indicating that the sugar-sugar bond is more prone for collision-induced dissociation (CID) than the hexose-coumaroyl bond. Next, another metabolic shift of +6C, +10H and +5O from *m/z* 727 to *m/z* 889 (“A3”) was detected by *mzGroupAnalyzer*, which indicates a hexosylation reaction. Indeed, MS^2^ fragmentation scans again showed peaks at *m/z* 287 and *m/z* 449, as well as *m/z* 727 itself in the spectrum, proving our assumptions that CID is happening in both positions, 3-O as well as 5-O. From *m/z* 889 to *m/z* 975, a malonylation step was detected. In the product ion scans, the fragment *m/z* 727 is again present, as well as a new peak at *m/z* 535, with *m/z* 449 nearly disappearing. *m/z* 975 has been reported as molecule “A5” before [33], and coincides with all our findings both in the metabolic route as well as in the MS^2^ spectra. The last step in this reaction list was detected to be a coumaroylation from *m/z* 975 to *m/z* 1121. Again, fragment 535 is found in the MS^2^ scan, while now a higher peak, *m/z* 873, emerges. We assume this peak is the structure of *m/z* 727 with another coumaroyl group in position 2 of the xylose, as this position tends to carry further groups. The peaks at *m/z* 1077 and *m/z* 931 in the MS^2^ product ion scan of *m/z* 1121 and *m/z* 975, respectively, correspond to a decarboxylation of the malonyl group in the native molecule. Furthermore the *m/z* 491 in these two MS^2^ product ion scans supports the decarboxylation reaction of *m/z* 535. Figure 6 shows additional MS^3^ product ion scans of the isolated MS^2^ product ions *m/z* 535, 873 and 1077. Both MS^2^ peaks *m/z* 535 and 873 result in the core cyanidin structure *m/z* 287 by undergoing MS^3^ fragmentation. *m/z* 1077, the putatively decarboxylized form of *m/z* 1121, yields *m/z* 491, as observed in the MS^2^ spectrum already, by CID-cleavage of the 3-O-glycosidic bond. Fragment *m/z* 873 arises again from the CID-cleavage of the glycosidic bond at 5-O. *m/z* 1017 is a putative structure generated by CID cleavage of an acetyl-group together with a water loss (−60 u). These findings lead us to propose *m/z* 1121 as compound “[Cy+2Glc+Xyl+2Cou+Mal]^+^”, or systematically, cyanidin 3-O-[2″-O-(6′″-O-(p-coumaroyl) xylosyl) 6″-O-(p-O-(glucosyl)-p-coumaroyl) glucoside] 5-O-(6″″-O-malonyl) glucoside. The resulting putative structure is shown in Figure 5.

*mzGroupAnalyzer* detected a differential appearance of various anthocyanidins (see Table 1) during the different time points. *m/z* 287 was detected in all time points, *m/z* 449 after 2 days, and 595, 727, 889, 975 and 1121 only after 4 days of oxidative stress. Following this strategy, 15 new compounds in *Arabidopsis thaliana* could be proposed by investigating the *mzGroupAnalyzer* pathway suggestions together with their product ion spectra *m/z*. Putative compounds with their *mzGroupAnalyzer*- predicted sum formulas, the corresponding exact mass as well as dominant MS^2^ product ion fragments are summarized in Table 1.

**Figure 7:**
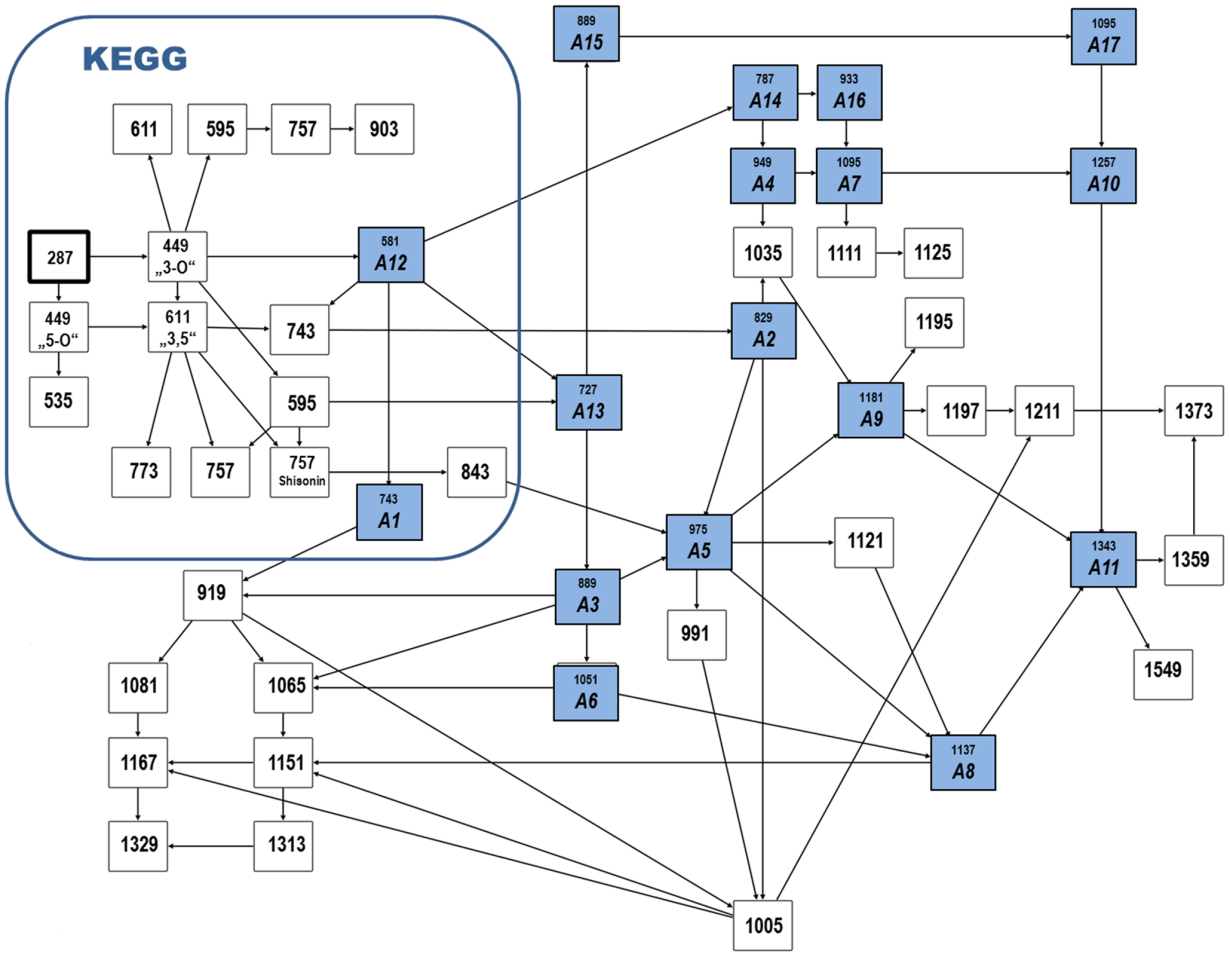
A proposed network of the detected anthocyanin family featuring putatively novel compounds as well as known structures including the KEGG pathway of anthocyanin biosynthesis [38], [44]. Only compounds from the KEGG anthocyanin pathway are depicted, for which a suitable precursor mass was found in the data. Exact masses, sum formulas, and main MS^2^ fragments of the new compounds are compiled in Table 1; reconstructed structures together with MS^2^ scans are in the supporting information. The network was created with VANTED [45], [46].

Using these new substances in combination with confirmed compounds from the literature, a network of the anthocyanin family starting with the KEGG pathway was reconstructed (Figure 7). Product ion scans of the new compounds and their reconstructed structures can be found in the Figure S2.

## Conclusions

In this study, we showed that the application of *mzGroupAnalyzer* – a novel algorithm for untargeted identification of chemical modifications in metabolome data – on time-dependent, high-throughput LC-Orbitrap-FT-MS metabolomic profiles can give new insights into biochemical pathways, and combined with MS^n^ scans has the power to validate known compounds and predict new structures. Attempts at assigning sum formulas to highly resolved metabolomic data have been done before and have proven to be very fruitful [34], [35], yet it is clear that those approaches ultimately have to be automatized. The visualization of the elemental composition of metabolites on a van Krevelen diagram is useful for recognition of metabolic patterns, which can point to a structural similarity between those molecules.

The unambiguous structural assignment and stereochemical elucidation of a compound is always a time-intensive process and demands further validation from other analytical platforms, such as NMR [7]. In our approach, we circumvent some of these issues by capturing metabolic intermediates of a compound in the data set, thus creating an additional layer of information. The complementary analysis of product ion spectra associated with the predicted chemical modifications of the precursor *m/z*-ratios introduces novel aspects for structural elucidation of unknown metabolites, and enables further validation. While the issues of unambiguous sum formula annotation in high-resolution LC-MS metabolomics still remain, and further application and validation of other analytical techniques are needed, *mzGroupAnalyzer* proves to be a convenient tool for tracking metabolic changes, thus sum formulas, and inferring metabolic pathways from time-series data, leading to the prediction of entirely novel, hitherto undescribed compounds. Furthermore, *mzGroupAnalyzer* is able to handle time-series data and is thus able to identify time-dependent chemical modifications. Finally, *mzGroupAnalyzer* is connected with the *Pathway Viewer* which plots corresponding pathways in a user friendly way.

Several strategies will be implemented in future to further improve the algorithm. First, the transformation frequency will be used in future to rank sum formulas corresponding to the same *m/z* feature. Secondly, more strict sum formula filtering criteria will be applied in selecting correct sum formulas from *m/z* features [36]. Thirdly, more reaction rules, and how these rules are connected in a pathway, will be learned from metabolic pathway databases ([37], [38] for KEGG and MetaCyc).

## Supporting Information

Figure S1 “S1 mzGA tutorial.pptx”; tutorial for mzGroupAnalyzer in pptx format.
(PPTX)

Figure S2 “S2 MS2 putative cyanidins.pptx”; recorded MS2 product ion scans of the putative new cyanins in pptx format.
(PPTX)

Table S1 Rules file for chemical and biochemical transformations.
(XLSX)

## References

[1] Fiehn O, Kopka J, Dormann P, Altmann T, Trethewey RN, et al (2000) Metabolite profiling for plant functional genomics. Nature Biotechnology 18: 1157–1161. 10.1038/81137 11062433

[2] Soga T (2007) Capillary electrophoresis-mass spectrometry for metabolomics. Methods Mol Biol 358: 129–137. 17035684 10.1007/978-1-59745-244-1_8

[3] Weckwerth W (2011) Unpredictability of metabolism-the key role of metabolomics science in combination with next-generation genome sequencing. Analytical and Bioanalytical Chemistry 400: 1967–1978. 21556754 10.1007/s00216-011-4948-9 PMC3098350

[4] Scherling C, Roscher C, Giavalisco P, Schulze ED, Weckwerth W (2010) Metabolomics unravel contrasting effects of biodiversity on the performance of individual plant species. Plos One 5: e12569. 20830202 10.1371/journal.pone.0012569 PMC2935349

[5] Mari A, Lyon D, Fragner L, Montoro P, Piacente S, et al (2013) Phytochemical composition of L. analyzed by an integrative GC-MS and LC-MS metabolomics platform. Metabolomics 9: 599–607. 23678344 10.1007/s11306-012-0473-x PMC3651535

[6] Doerfler H, Lyon D, Nagele T, Sun X, Fragner L, et al (2013) Granger causality in integrated GC-MS and LC-MS metabolomics data reveals the interface of primary and secondary metabolism. Metabolomics: Official journal of the Metabolomic Society 9: 564–574. 23678342 10.1007/s11306-012-0470-0 PMC3651536

[7] Dunn WB, Erban A, Weber RJM, Creek DJ, Brown M, et al.. (2012) Mass appeal: metabolite identification in mass spectrometry-focused untargeted metabolomics. Metabolomics.

[8] Olsen JV, de Godoy LM, Li G, Macek B, Mortensen P, et al (2005) Parts per million mass accuracy on an Orbitrap mass spectrometer via lock mass injection into a C-trap. Molecular & cellular proteomics: MCP 4: 2010–2021. 16249172 10.1074/mcp.T500030-MCP200

[9] Kind T, Fiehn O (2010) Advances in structure elucidation of small molecules using mass spectrometry. Bioanalytical reviews 2: 23–60. 21289855 10.1007/s12566-010-0015-9 PMC3015162

[10] Weckwerth W (2011) Green systems biology - From single genomes, proteomes and metabolomes to ecosystems research and biotechnology. Journal of Proteomics 75: 284–305. 21802534 10.1016/j.jprot.2011.07.010

[11] Weckwerth W, Morgenthal K (2005) Metabolomics: from pattern recognition to biological interpretation. Drug Discov Today 10: 1551–1558. 16257378 10.1016/S1359-6446(05)03609-3

[12] van der Greef J, Hankemeier T, McBurney RN (2006) Metabolomics-based systems biology and personalized medicine: moving towards n = 1 clinical trials? Pharmacogenomics 7: 1087–1094. 17054418 10.2217/14622416.7.7.1087

[13] Mittler R, Vanderauwera S, Gollery M, Van Breusegem F (2004) Reactive oxygen gene network of plants. Trends in plant science 9: 490–498. 15465684 10.1016/j.tplants.2004.08.009

[14] Croteau R, Kutchan TM, Lewis NG (2000) Natural Products (Secondary Metabolites). Biochemistry and Molecular Biology of Plants: 1250–1318.

[15] Lee KW, Lee HJ, Lee CY (2004) Vitamins, phytochemicals, diets, and their implementation in cancer chemoprevention. Crit Rev Food Sci Nutr 44: 437–452. 15615427 10.1080/10408690490886674

[16] Sun X, Weckwerth W (2012) COVAIN: a toolbox for uni- and multivariate statistics, time-series and correlation network analysis and inverse estimation of the differential Jacobian from metabolomics covariance data. Metabolomics: Official journal of the Metabolomic Society 8: 81–93.

[17] Kind T, Fiehn O (2006) Metabolomic database annotations via query of elemental compositions: mass accuracy is insufficient even at less than 1 ppm. BMC Bioinformatics 7: 234. 16646969 10.1186/1471-2105-7-234 PMC1464138

[18] Quenzer T (2002) Automated accurate mass analysis using FTICR mass spectrometry, Proceedings of the 50th Annual Conference on Mass Spectrometry and Allied Topics, Orlando, FL.

[19] Kimura M, Yamamoto YY, Seki M, Sakurai T, Sato M, et al (2003) Identification of Arabidopsis genes regulated by high light-stress using cDNA microarray. Photochemistry and Photobiology 77: 226–233. 12785063 10.1562/0031-8655(2003)077<0226:ioagrb>2.0.co;2

[20] Dong CH, Zolman BK, Bartel B, Lee BH, Stevenson B, et al (2009) Disruption of Arabidopsis CHY1 reveals an important role of metabolic status in plant cold stress signaling. Molecular plant 2: 59–72. 19529827 10.1093/mp/ssn063 PMC2639738

[21] Huang X, Li Y, Zhang X, Zuo J, Yang S (2010) The Arabidopsis LSD1 gene plays an important role in the regulation of low temperature-dependent cell death. The New phytologist 187: 301–312. 20456049 10.1111/j.1469-8137.2010.03275.x

[22] Nishiyama Y, Yamamoto H, Allakhverdiev SI, Inaba M, Yokota A, et al (2001) Oxidative stress inhibits the repair of photodamage to the photosynthetic machinery. The EMBO journal 20: 5587–5594. 11598002 10.1093/emboj/20.20.5587 PMC125664

[23] Asada K (2006) Production and scavenging of reactive oxygen species in chloroplasts and their functions. Plant physiology 141: 391–396. 16760493 10.1104/pp.106.082040 PMC1475469

[24] Apel K, Hirt H (2004) Reactive oxygen species: metabolism, oxidative stress, and signal transduction. Annual review of plant biology 55: 373–399. 10.1146/annurev.arplant.55.031903.141701 15377225

[25] Hernandez I, Alegre L, Van Breusegem F, Munne-Bosch S (2009) How relevant are flavonoids as antioxidants in plants? Trends in plant science 14: 125–132. 19230744 10.1016/j.tplants.2008.12.003

[26] Seyoum A, Asres K, El-Fiky FK (2006) Structure-radical scavenging activity relationships of flavonoids. Phytochemistry 67: 2058–2070. 16919302 10.1016/j.phytochem.2006.07.002

[27] Bashandy T, Taconnat L, Renou JP, Meyer Y, Reichheld JP (2009) Accumulation of flavonoids in an ntra ntrb mutant leads to tolerance to UV-C. Mol Plant 2: 249–258. 19825611 10.1093/mp/ssn065

[28] Kimura M, Yamamoto YY, Seki M, Sakurai T, Sato M, et al (2003) Identification of Arabidopsis genes regulated by high light-stress using cDNA microarray. Photochem Photobiol 77: 226–233. 12785063 10.1562/0031-8655(2003)077<0226:ioagrb>2.0.co;2

[29] Wu Z, Rodgers RP, Marshall AG (2004) Two- and three-dimensional van krevelen diagrams: a graphical analysis complementary to the kendrick mass plot for sorting elemental compositions of complex organic mixtures based on ultrahigh-resolution broadband fourier transform ion cyclotron resonance mass measurements. Analytical chemistry 76: 2511–2516. 15117191 10.1021/ac0355449

[30] van Krevelen DW (1950) Graphical-statistical method for the study of structure and reaction processes of coal. Fuel: 269–284.

[31] Kai K, Takahashi H, Saga H, Ogawa T, Kanaya S, et al (2011) Metabolomic characterization of the possible involvement of a Cytochrome P450, CYP81F4, in the biosynthesis of indolic glucosinolate in Arabidopsis. Plant Biotechnology 28: 379–385.

[32] Reemtsma T (2009) Determination of molecular formulas of natural organic matter molecules by (ultra-) high-resolution mass spectrometry: status and needs. Journal of chromatography A 1216: 3687–3701. 19264312 10.1016/j.chroma.2009.02.033

[33] Tohge T, Nishiyama Y, Hirai MY, Yano M, Nakajima J, et al (2005) Functional genomics by integrated analysis of metabolome and transcriptome of Arabidopsis plants over-expressing an MYB transcription factor. The Plant journal: for cell and molecular biology 42: 218–235. 15807784 10.1111/j.1365-313X.2005.02371.x

[34] Giavalisco P, Hummel J, Lisec J, Inostroza AC, Catchpole G, et al (2008) High-resolution direct infusion-based mass spectrometry in combination with whole 13C metabolome isotope labeling allows unambiguous assignment of chemical sum formulas. Analytical chemistry 80: 9417–9425. 19072260 10.1021/ac8014627

[35] Rogers S, Scheltema RA, Girolami M, Breitling R (2009) Probabilistic assignment of formulas to mass peaks in metabolomics experiments. Bioinformatics 25: 512–518. 19095699 10.1093/bioinformatics/btn642

[36] Kind T, Fiehn O (2007) Seven Golden Rules for heuristic filtering of molecular formulas obtained by accurate mass spectrometry. BMC Bioinformatics 8: 105. 17389044 10.1186/1471-2105-8-105 PMC1851972

[37] Karp PD, Riley M, Paley SM, Pellegrini-Toole A (2002) The MetaCyc Database. Nucleic acids research 30: 59–61. 11752254 10.1093/nar/30.1.59 PMC99148

[38] Kanehisa M (2002) The KEGG database. Novartis Foundation symposium 247: : 91–101; discussion 101–103, 119–128, 244–152. 12539951

[39] Saito N, Tatsuzawa F, Nishiyama A, Yokoi M, Shigihara A, et al (1995) Acylated cyanidin 3-sambubioside-5-glucosides in Matthiola incana. Phytochemistry 38: 1027–1032. 7766384 10.1016/0031-9422(94)00659-h

[40] Bloor SJ, Abrahams S (2002) The structure of the major anthocyanin in Arabidopsis thaliana. Phytochemistry 59: 343–346. 11830144 10.1016/s0031-9422(01)00460-5

[41] Kasai HF, Saito N, Honda T (2011) Structural features of polyacylated anthocyanins using matrix-assisted laser desorption/ionization and electrospray ionization time-of-flight mass spectrometry. Rapid communications in mass spectrometry: RCM 25: 1051–1060. 21452382 10.1002/rcm.4956

[42] March RE, Miao X-S (2004) A fragmentation study of kaempferol using electrospray quadrupole time-of-flight mass spectrometry at high mass resolution. International Journal of Mass Spectrometry 231: 157–167.

[43] Abad-Garcia B, Garmon-Lobato S, Berrueta LA, Gallo B, Vicente F (2009) A fragmentation study of dihydroquercetin using triple quadrupole mass spectrometry and its application for identification of dihydroflavonols in Citrus juices. Rapid communications in mass spectrometry: RCM 23: 2785–2792. 19653204 10.1002/rcm.4182

[44] Kanehisa M, Goto S, Sato Y, Kawashima M, Furumichi M, et al (2014) Data, information, knowledge and principle: back to metabolism in KEGG. Nucleic acids research 42: D199–205. 24214961 10.1093/nar/gkt1076 PMC3965122

[45] Junker BH, Klukas C, Schreiber F (2006) VANTED: a system for advanced data analysis and visualization in the context of biological networks. BMC bioinformatics 7: 109. 16519817 10.1186/1471-2105-7-109 PMC1413562

[46] Rohn H, Junker A, Hartmann A, Grafahrend-Belau E, Treutler H, et al (2012) VANTED v2: a framework for systems biology applications. BMC systems biology 6: 139. 23140568 10.1186/1752-0509-6-139 PMC3610154

